# Cyberism Perception–Interaction Layer: A Framework for AI-Enabled Sensing Between Humans and Cyberspace

**DOI:** 10.3390/s26196122

**Published:** 2026-09-27

**Authors:** Lei Zhou, Jianguo Ding, Huansheng Ning

**Affiliations:** 1 School of Information Engineering, Xinjiang Institute of Engineering, Urumqi 830023, China; d202510481@xs.ustb.edu.cn; 2 School of Computer and Communication Engineering, University of Science and Technology Beijing, Beijing 100083, China; 3 School of Computing, Blekinge Institute of Technology, 37179 Karlskrona, Sweden

**Keywords:** Cyberism, AI sensors, AIoT sensing, edge AI, perception–interaction layer, wearable sensing, haptic feedback, multimodal sensor fusion, human–cyberspace relationships

## Abstract

**Highlights:**

**What are the main findings?**

- The paper frames AI-enabled sensing as a human–cyberspace relation rather than only a signal-processing pipeline.
- Three sub-domains are proposed: exteroceptive, interoceptive, and machine-native cyber-sensing, with an integrated crosswalk showing their distinct evaluation burdens.

**What are the implications of the main findings?**

- AI-sensor evaluation should pair technical metrics with appropriately operationalized human-centered measures of perceptual coherence, interoceptive agency, and perceptual explainability.
- The Cyberism framework gives intelligent-sensor research a design agenda that links edge AI sensing with human autonomy, oversight, and equity in cyber-mediated sensing.

**Abstract:**

Artificial intelligence (AI) and sensing technologies increasingly mediate how humans encounter physical, bodily, and digital environments. Yet intelligent-sensor frameworks still emphasize accuracy, latency, power, and robustness while giving less attention to the perceptual relation created between humans and cyberspace. This conceptual and integrative review uses directed conceptual synthesis, purposive literature selection, contrastive case analysis, and negative-case testing to develop the perception–interaction layer of Cyberism for AI-enabled sensing. The synthesis distinguishes exteroceptive cyber-sensing, which extends or reconstructs familiar external senses; interoceptive cyber-sensing, which externalizes and interprets bodily states; and machine-native cyber-sensing, which translates modalities such as radar and LiDAR into humanly actionable perception. These sub-domains are mapped, on a many-to-many basis, to three proposed evaluation constructs that differ in measurement maturity: perceptual coherence, interoceptive agency, and perceptual explainability. Three hardware-constant examples, an integrated worked crosswalk, and an engineering signal-chain comparison show how the same sensing hardware can require different interfaces, monitoring provisions, and human outcome measures when its perceptual role changes. The framework remains a proposal requiring empirical validation, but it contributes an auditable method for linking AI sensor pipelines to uncertainty communication, feedback-induced distribution change, appropriate reliance, human agency, oversight, and differential performance.

## 1. Introduction

AI-enabled sensing is changing the meaning of perception. Cameras, LiDARs, radars, wearable biosensors, electronic skins, acoustic arrays, and chemical sensors no longer merely collect signals. Increasingly, they classify, infer, predict, and feed back information in real time. They are embedded in bodies, vehicles, homes, hospitals, factories, and urban infrastructure. Edge computing and edge intelligence increasingly support this shift by moving sensing, inference, and response closer to devices and users [1,2]. As a result, sensing is becoming one of the principal interfaces between humans and cyberspace.

The dominant language used to describe this transformation is still technical. Intelligent sensors are evaluated by accuracy, resolution, latency, bandwidth, energy efficiency, reliability, robustness, and cost. These criteria are necessary. An unreliable sensor cannot support trustworthy inference, and an energy-intensive edge device cannot scale in distributed AIoT systems [1,2]. Recent AI-sensor research includes work on neuromorphic tactile perception for robotics, linking physical transduction with biologically inspired computation [3]. However, a purely metric-centered view leaves out a basic question: when a sensing system observes the world, interprets the body, or translates a machine-native signal, what kind of perceptual relation does it create between humans and cyberspace?

This question matters because sensing is not a neutral front end. It selects what becomes visible, measurable, actionable, and governable, thereby mediating how users encounter the world through technology [4,5]. In an AI-assisted wearable, physiological signals become external dashboards and recommendations. In a tactile interface, force, vibration, and pressure are remapped into designed bodily experience. In autonomous driving, radar and LiDAR create perceptual channels that humans do not naturally possess, and AI translates those channels into warnings, decisions, or explanations [6,7,8]. In each case, sensing is not only a technical operation but also a reconfiguration of embodied experience, agency, and trust.

The argument developed here begins from three premises that can be assessed without accepting Cyberism as a doctrine. First, an AI sensor system can transform a physical or physiological signal into a human-facing perceptual object rather than merely produce a machine-readable estimate. Second, different signal-to-perception transformations expose different human failure modes, including action–feedback conflict, displacement of bodily judgment, and opaque reliance on machine-native evidence. Third, those human failure modes require evaluation criteria beyond device and model performance. A classification is useful only when it changes a design choice, a measurement choice, or an oversight requirement; otherwise it adds terminology without analytic value.

Cyberism is introduced after these premises as the organizing framework rather than as their proof. It has recently been proposed as a doctrine for studying fundamental relationships between humans and cyberspace [9,10]. It builds on a four-layer disciplinary framework and earlier cyber-physical-social-thinking (CPST) research that treats cyberspace not as a detached virtual layer but as a space intertwined with physical, social, and thinking spaces [11,12]. For AI-enabled sensing, its useful role is to name and organize how technological mediation changes the relation between human beings and the cyber-enabled world. The framework must still be compared with established theories and tested against cases in which its categories fail to change design or evaluation.

Cyberism enters an established discussion of technological mediation. Ihde’s postphenomenology distinguishes embodiment, hermeneutic, alterity, and background relations, while Verbeek extends this relational account toward the ethical and behavioral mediation of technology [4,5]. These categories explain whether a technology becomes incorporated into bodily action, represents a world that users must interpret, appears as a quasi-other, or shapes experience from the background. An AI sensor system can participate in several such relations at once: a haptic device may be embodied, a health dashboard may be hermeneutic, and an autonomous system may operate as both alterity and background.

Related traditions emphasize different aspects of technologically altered perception. Sensory-substitution and human-augmentation research examines how information is remapped across modalities or extends human capability [13,14]. Soma design foregrounds first-person bodily experience and the design process through which users learn to perceive with interactive technologies [15]. Cognitive-offloading research, in turn, analyzes how external artifacts reduce or redistribute internal information-processing demands [16]. These traditions provide essential accounts of mediation, embodiment, augmentation, and reliance.

The three Cyberism sub-domains are therefore intended as a complementary axis rather than a replacement taxonomy. They classify AI-enabled sensing by the relation between signal origin and perceptual transformation: extension of a familiar external sense, externalization of a bodily state, or translation of a machine-native modality. This axis connects philosophical mediation to sensor architecture, human evaluation, and questions of governance. Its proposed contribution is not the general claim that technology mediates perception, but an operational crosswalk for specifying what an AI sensor makes perceptible, how that perception is evaluated, and which forms of agency and oversight are at stake.

The specific focus of this paper is the perception–interaction layer of Cyberism. This layer is the entry point at which humans and cyberspace meet through sensing, feedback, and action. The paper argues that the layer can provide a compact framework for AI-enabled sensing by classifying intelligent sensing into three sub-domains: exteroceptive, interoceptive, and machine-native cyber-sensing. These labels are not intended to invoke classical cybernetics in the Wienerian control-theory sense or to replace engineering taxonomies based on sensor type or algorithmic architecture. Rather, they ask a complementary question: what human perceptual channel is being extended, externalized, or newly created?

The contribution of this paper is fourfold. First, it translates three independently assessable premises into a design-oriented framework for AI-enabled sensing. Second, it defines three sub-domains and a many-to-many matrix connecting them to perceptual coherence, interoceptive agency, and perceptual explainability. Third, it demonstrates the framework through hardware-constant examples and an engineering signal-chain comparison rather than classifying systems by hardware names alone. Fourth, it proposes monitoring, validation, and reporting practices for feedback-induced distribution change, oversight, and differential performance. The goal is to give sensor engineering a human-centered conceptual grammar precise enough to generate testable and falsifiable questions. Figure 1 situates the perception–interaction layer within the broader Cyberism framework.

Prior Cyberism publications establish the doctrine, its four-layer structure, and related CPST terminology. The distinct contribution of the present review is to operationalize the perception–interaction layer for AI-enabled sensing through a three-sub-domain taxonomy, a many-to-many construct matrix, an integrated worked crosswalk, hardware-constant contrasts, auditable design records, and a falsifiable empirical validation protocol.

The paper does not introduce a new sensor, dataset, model, psychometric instrument, or empirical effect. Its contribution is an operational crosswalk whose value should be judged by whether it changes failure analysis, measurement selection, or oversight beyond established sensor-type or task taxonomies. The integrated case is an analytical demonstration of possible decision divergence, not evidence of empirical incremental validity.

The upper three layers in Figure 1 provide contextual positioning only. They are not analyzed or validated in this review.

## 2. Conceptual Approach

This article is a conceptual and integrative review rather than an experimental report or an exhaustive evidence synthesis. The method combines directed conceptual synthesis, domain mapping, contrastive case analysis, and negative-case testing, consistent with established guidance for constructing conceptual frameworks and conducting integrative literature reviews [17,18]. It is not a systematic or scoping review and does not support prevalence estimates or pooled effect claims. The unit of analysis is not a single sensor, algorithm, or dataset; it is the perceptual relation produced when sensors, AI models, human users, and cyber-physical environments are coupled.

Literature was selected purposively across six bodies of scholarship needed to build and challenge the framework: sensor and edge-AI engineering; Cyberism and cyber-physical-social-thinking research; postphenomenology and embodied cognition; sensory substitution, augmentation, and soma design; human factors, situation awareness, explainable AI, and trust in automation; and clinical, ethical, and equity studies of AI-enabled sensing. Priority was given to foundational works, peer-reviewed reviews, benchmark or systems papers that establish engineering capabilities and failure modes, and empirical studies that report human or clinical consequences. The three cases were selected by contrastive logic: each represents a different signal origin and perceptual transformation, has a mature technical literature, and exposes a distinct limitation of performance-only evaluation. The cases are therefore analytic demonstrations, not estimates of prevalence, effect size, or generality. The integrated crosswalk below recombines these documented signal paths in one human decision context as a stress test of the mapping protocol, not as a fourth literature case or a representative deployed system.

Five publications coauthored by H.N. were retained because they document the provenance of Cyberism, its four-layer structure, related CPST terminology, and an earlier selective-sensing model [9,10,11,12,19]. They are not treated as independent confirmation of the present taxonomy, constructs, or human outcomes. Support for those claims is instead sought in the adjacent engineering, human-factors, clinical, and philosophical literatures, and the framework is explicitly exposed to negative cases and falsification criteria.

The synthesis proceeded in four steps. First, we identified the Cyberism concepts most relevant to sensing: cyberspace as a basic living space, the four-layer disciplinary structure, and the foundational role of perception and interaction. Second, we connected these concepts to established discussions in IoT, AIoT, and selective sensing, where sensors mediate the mapping between physical processes and cyber representations [19,20,21]. Third, we drew on ecological perception, phenomenology (as a practical lens for analyzing first-person experience of sensor feedback), extended cognition, postphenomenology, and philosophy of technology to clarify why sensing should be analyzed as an embodied relation rather than as information input alone [4,5,22,23,24]. Fourth, we selected three representative AI sensor cases: haptic feedback, wearable health sensing, and autonomous driving perception. They correspond to three different forms of human perceptual transformation.

Because these literatures answer different questions, the synthesis did not treat every source as the same kind of evidence. Foundational philosophical and theoretical works were used to define relations among body, environment, artifact, and representation; they were not used to support claims about contemporary sensor performance. Engineering surveys, systems papers, and benchmark publications were used to establish signal chains, technical capabilities, and recognized failure conditions; they were not assumed to demonstrate human benefit. Human-factors and clinical studies were used where the argument concerns behavior, trust, workload, anxiety, or treatment-related consequences. Ethics and governance reviews were used to identify recurring normative concerns, not to infer the magnitude of a technical disparity. Keeping these evidentiary roles separate reduces the risk of moving silently from a conceptual analogy to an empirical conclusion.

Selection also followed a relevance rule. A work was retained when it contributed directly to at least one of four analytic functions: defining a perceptual relation, documenting an AI sensor capability or limitation, supplying a human-centered evaluation construct, or showing a consequence of sensing for users or affected groups. Foundational works were not restricted by publication date because their role is conceptual lineage. Technical and empirical sources were updated through July 2026 to include recent work relevant to AI-enabled sensing, wearables, haptics, autonomous perception, and sensor equity. Bibliographic metadata, DOI resolution, and official source landing pages were rechecked before compilation of this revision. This verification establishes source identity and citation accuracy; it does not convert the purposive synthesis into an exhaustive review.

For transparency, literature discovery used targeted keyword searches in web-indexed scholarly sources and direct searches of IEEE Xplore, ACM Digital Library, PubMed, and official publisher or DOI landing pages; backward and forward citation chaining was used to identify foundational and adjacent work. The working discovery window for technical and empirical literature was 1 January 2010 through 31 July 2026; earlier sources were retained when they supplied conceptual lineage. Search strings combined terms for AI-enabled sensing, human–AI interaction, haptics, wearables, interoception, autonomous perception, sensor fusion, uncertainty, fairness, and governance.

The process was author-led and purposive rather than a duplicate-screening systematic review. L.Z. prepared the initial source and concept extraction; J.D. and H.N. reviewed evidence-role assignments, boundary cases, and high-impact claims. Disagreements were resolved through discussion; when consensus was not reached, the claim was narrowed, marked as a proposal, or excluded. The retained record stores the source key, DOI or official landing page, evidence role, extracted claim, boundary note, and decision/status. The stopping rule was reached when additional targeted searches no longer supplied a new signal-to-perceptual transformation, failure mode, evaluation construct, or governance consequence for the three selected cases.

For each retained source, the conceptual extraction focused on the signal source, the AI operation, the human-facing representation, the expected user action, the principal failure mode, and the level of evidence. This common matrix allowed work from otherwise separate fields to be compared without claiming that their constructs are interchangeable. For example, a model-calibration paper can identify where uncertainty enters a sensing pipeline, but a human-factors study is still needed to determine whether a displayed confidence value produces appropriate reliance. Conversely, a trust study can show a behavioral response to an interface, but it cannot establish the physical accuracy of the underlying sensor. The framework is built from the relation between these evidence types rather than from substitution of one for another.

The synthesis also considered boundary and negative cases. A sensor that records data solely for machine-to-machine control, with no reasonably identifiable human recipient or affected decision, does not automatically instantiate the human-centered relation analyzed here. A familiar camera does not automatically count as exteroceptive extension if its output is transformed into a machine-only latent representation; classification depends on what is eventually made available for human perception or action. Likewise, a physiological sensor is not interoceptive merely because it is attached to the body: a motion sensor used as a gesture controller may function exteroceptively, whereas the same device used to infer sleep or fatigue may function interoceptively. These tests guard against classifying systems by hardware names alone.

Finally, the review distinguishes three levels of claim. A descriptive claim states what a source reports about a device, model, interface, or observed outcome. An analytic claim maps that evidence into the proposed taxonomy. A normative claim states what designers or institutions should evaluate or preserve. The latter two are contributions of this review and are labeled as proposals rather than established effects. No numerical result is introduced unless it is reported by a cited source, and the paper does not alter or pool source data.

### Review Quality and Reflexive Controls

To prevent Cyberism from functioning as its own validation criterion, each central premise was audited separately from the doctrine. For every premise, we recorded the non-Cyberism evidence base, the evidentiary role of each source, a plausible alternative classification, at least one negative or boundary case, and the design or measurement consequence that would remain after removing Cyberism terminology. The resulting premise-to-evidence audit is reported in Appendix A, Table A1. Cyberism publications were used only to document conceptual provenance and were not counted as independent support for the proposed taxonomy or human outcomes.

Audit outcome: P1 was retained but narrowed to systems with an identifiable human-facing perceptual object; P2 was retained as a falsifiable analytic premise; and P3 was retained as a proposed normative and measurement requirement. No premise was excluded, because each changed at least one design, measurement, or oversight record. None was treated as empirically validated by Cyberism publications.

The review was evaluated against four internal quality checks. First, each central proposition was assigned an evidence role: conceptual lineage, engineering capability or limitation, human-factor or clinical consequence, or normative recommendation. Second, the classification was stress-tested with hardware-constant contrasts, hybrid cases, and cases outside the human-facing scope. Third, self-citations were separated from independent support: the H.N.-coauthored publications document the provenance of Cyberism, whereas claims about haptics, wearables, automation, fairness, and human outcomes are supported by adjacent literatures. Fourth, a claim was retained only when the proposed category changed a design choice, measurement choice, or oversight requirement.

These checks address a known logical risk. Cyberism organizes the synthesis but cannot serve as its own validation criterion. The premises about human-facing signal transformation, distinct failure modes, and the need for human-centered evaluation must remain intelligible and challengeable when the Cyberism label is removed. Accordingly, the review treats the stand-alone toolkit and the broader doctrine as separable layers and records the absence of empirical construct validation as a limitation rather than as support for the framework.

The mapping protocol used in this paper asks four questions for each sensing system:

1. What is the primary signal source: external environment, internal bodily state, or machine-native modality?
2. What human perceptual channel is affected: a classical external sense, bodily self-awareness, or a newly translated perceptual dimension?
3. What is the main AI function: enhancement, externalization, translation, prediction, or explanation?
4. What human–cyberspace relation is created: substitution, reconfiguration, offloading, co-perception, or perceptual governance?

Operationally, the protocol can be used as a three-step decision flow: identify the signal source, identify the perceptual object offered to the user, and then classify the dominant mediation as extension, externalization, or translation. The more detailed questions above make this flow auditable when a system is hybrid.

To make the mapping reproducible, this paper treats a sensing system as belonging to the sub-domain that dominates the user’s perceptual relation, not simply the sub-domain suggested by the physical sensor component. For example, an inertial measurement unit embedded in a smart ring can support exteroceptive gesture interaction or interoceptive sleep interpretation depending on how its output is made meaningful to the user. Likewise, the same in-cabin RGB or near-infrared camera stream can support gaze-based interaction or infer a driver’s fatigue and attention state. The classification therefore follows the direction of mediation: whether the system extends an already familiar external sense, externalizes a bodily state, or translates a nonhuman sensing channel into a humanly actionable form.

The protocol also distinguishes the *signal object* from the *perceptual object*. The signal object is what the sensor captures, such as acceleration, light intensity, point-cloud geometry, skin conductance, or pressure. The perceptual object is what the human is invited to experience, such as balance, fatigue, obstacle proximity, road risk, pain, stress, or texture. AI sensor systems may fail at the level of use even when this translation is technically plausible. A high-confidence classification may still be perceived as arbitrary if the user cannot connect it to bodily feeling or environmental evidence, while a modest signal estimate may remain useful when its uncertainty, context, and action implication are communicated clearly [4,25].

Finally, the method treats human feedback as part of the sensing system. In many AIoT settings, users label events, adjust thresholds, change behavior after receiving prompts, or physically reposition devices. These actions can alter later covariates, labels, missingness, and exposure to events. A Cyberism analysis therefore examines not only the first-pass inference but also the adaptive loop between sensor output, human interpretation, behavioral response, and subsequent measurement. This loop is particularly important for wearables and autonomous systems, where repeated feedback can calibrate trust and improve fit, or create misuse, over-reliance, and feedback-induced distribution change [26,27].

This protocol is intentionally lightweight. It does not replace quantitative evaluation, clinical validation, or autonomous-driving benchmarks. Instead, it adds a conceptual layer that can be used before, during, or after engineering evaluation. Before system design, it clarifies the perceptual role the system is expected to play. During development, it helps identify whether the system optimizes only signal-level performance or also human-level coherence. After deployment, it supports assessment of unintended effects such as dependence, inequality, opacity, or loss of agency, which are recurring concerns in automation research and AI ethics guidance [26,28].

The paper uses illustrative cases rather than exhaustive review. Haptic systems show that effective artificial touch depends on perceptual and task design as well as physical rendering [6]. Wearable health sensors show that increased measurement can support self-management while also raising reliability, interpretation, and behavioral concerns [7,29]. Autonomous driving perception shows that machine-native sensing requires translation into understandable explanations and intentions when humans supervise or share environments with autonomous systems [30,31]. Bibliographic details and source landing pages are verified separately from judgments about the strength or generalizability of the cited evidence.

## 3. The Cyberism Perception–Interaction Framework

The outputs of the conceptual approach are presented below as a positioning analysis, a three-part taxonomy, hardware-constant contrasts, an engineering translation, and a methodological agenda. They are conceptual synthesis results rather than outcomes of an experimental intervention.

### 3.1. Cyberism Positioning for AI-Enabled Sensing

Cyberism positions cyberspace as a basic living space in which human existence, communication, cognition, and action are increasingly organized [9,10]. For AI-enabled sensing, this means that a sensor is not merely an input device. It is a boundary object between human embodiment and cyber representation. It determines which signals enter cyberspace, how they are structured, and how they return to humans as feedback, recommendation, warning, or control.

Within the four-layer Cyberism framework, the perception–interaction layer is foundational because every higher cyber relation presupposes a perceptual interface [11]. Social coordination requires shared sensing and communication channels. Cognitive autonomy requires awareness of how signals become prompts, nudges, and decisions. Questions of meaning and identity arise only after humans have already experienced the world through cyber-mediated interfaces. Thus, perception is not a narrow technical layer; it is the entry condition for cyber life.

The independently assessable premises stated in the Introduction constrain this positioning. Cyberism supplies a name and organizing structure, not empirical confirmation. The taxonomy is supported only to the extent that it distinguishes failure analyses, interface provisions, human outcome measures, or oversight requirements better than established alternatives.

### 3.2. Minimum Stand-Alone Toolkit

The analytic instruments proposed here can be used without adopting the full Cyberism doctrine. A minimum stand-alone toolkit consists of five records: (1) a signal-object and perceptual-object description; (2) a dominant and secondary sub-domain classification; (3) a human-facing transformation record that states the intended action; (4) a construct-to-measurement map that separates technical, behavioral, and self-report indicators; and (5) a degradation, equity, and oversight record. These records are operational tools for sensor and human-factors studies, not tests of whether Cyberism is ontologically correct.

By contrast, the broader doctrine contains ontological and disciplinary assumptions, including cyberspace as a basic living space and the four-layer hierarchy. Those assumptions provide interpretive context, but they are not prerequisites for using the toolkit. A researcher may therefore test the three-sub-domain taxonomy and the three evaluation constructs while rejecting, suspending, or simply not addressing the broader doctrine. The framework’s empirical value should be judged by whether these instruments improve classification, measurement selection, or oversight decisions beyond an established sensor or task description.

Table 1 positions the Cyberism perception–interaction layer against five adjacent bodies of work. Sensor engineering supplies the technical substrate; postphenomenology explains structures of mediation; sensory substitution and augmentation examine changed perceptual capability; soma design and embodied HCI foreground felt experience; and AI ethics and governance address normative consequences [4,5,13,14,15,28]. Cyberism draws on all five, but adds a signal-to-perceptual-transformation axis designed specifically for AI-enabled sensing.

### 3.3. Relations to Adjacent Concepts and Boundary Conditions

Adjacent traditions answer different questions when AI inference is inserted into a sensing loop. Postphenomenology explains technological mediation through embodiment, hermeneutic, alterity, and background relations, while ecological, phenomenological, and extended-cognition accounts emphasize situated action, lived experience, and the redistribution of reasoning across people and artifacts. The Cyberism taxonomy complements these accounts by asking which signal-to-perception transformation is dominant and which evaluation burden follows [4,5,16,22,23,24].

Sensory-substitution research typically studies a remapping of information that is available through one channel into another, often with the aim of restoring or extending a familiar perceptual capacity. Machine-native cyber-sensing is different when the source modality, such as radar, thermal radiation, or hyperspectral structure, has no ordinary human equivalent and must first be translated into a task-relevant perceptual object. Both can involve learned mappings, but machine-native systems additionally require evidence about modality meaning, uncertainty, degradation, and the conditions under which the human should defer. A tactile layout for visual navigation is therefore a classic substitution or extension case; a tactile cue derived from radio-frequency occupancy is a machine-native translation case [13,14].

Soma design adds a first-person practice for exploring how feedback feels and is learned, but it does not by itself provide a universal measurement model. Human-automation, XAI, and situation-awareness research adds further constraints: automation research concerns reliance and misuse, XAI concerns model transparency or explanation techniques for a purpose and audience, and situation awareness concerns perception, comprehension, and projection in a dynamic environment. Perceptual explainability is narrower and interface-centered: a specified recipient must be able to connect sensed evidence, uncertainty, degradation, and likely action to a task-relevant judgment and contest or override it when authorized [15,26,27,32,33,34].

The taxonomy should not relabel every sensor interaction or imply consciousness in an AI model. Its scope is a coupled system in which computation transforms sensor signals into information that shapes human perception, judgment, or action. Its comparative claim is falsifiable: if the proposed categories do not lead to distinguishable failure analyses, measurement bundles, or oversight provisions beyond established sensor or task classifications, the taxonomy should be narrowed.

### 3.4. AI-Enabled Sensors as Human–Cyberspace Interfaces

AI-enabled sensors can be interpreted as a closed human–cyberspace loop. A transducer captures a physical, biological, or environmental signal. An AI model cleans, fuses, classifies, predicts, or interprets the signal. A cyber representation stores and communicates the result. A human-facing interface transforms the result into feedback, display, warning, recommendation, or automated actuation. Human behavior then modifies the environment or the body, producing new signals. This edge-to-human loop combines distributed sensing and inference with patterns of human use and reliance described in edge-intelligence and automation research [1,2,26]. Figure 2 summarizes this loop.

The loop reveals two points that are often hidden in technical descriptions. First, AI sensors do not simply perceive for humans; they often perceive with humans. Their outputs are integrated into user judgment, clinical interpretation, driving supervision, robotic control, or personal self-understanding. Second, the return path is as important as the sensing path. The same physiological signal may contribute to reassurance, anxiety, discipline, or dependence depending on how it is interpreted and fed back [27,29].

### 3.5. Feedback-Induced Distribution Change

The return path in Figure 2 can change the data on which later inferences depend. Ordinary distribution shift may arise from environmental change, sensor aging, replacement hardware, or a new user population. Feedback-induced change is more specific: an output causes a person or institution to alter behavior, placement, labeling, thresholds, or exposure, which then changes later covariates, labels, missingness, or event frequency. Performative-prediction research formalizes the broader case in which predictions influence the outcomes they are intended to predict and shows why repeated retraining does not by itself guarantee a stable or desirable result [35].

Wearables provide a direct sensing-loop example. A sleep or cardiac alert may change exercise, checking, help-seeking, device wear time, or sensor placement. The resulting stream is no longer sampled under the same behavioral conditions as the data that generated the alert. In a vehicle, a degradation warning can change gaze allocation or takeover readiness; a driver who repeatedly experiences nuisance warnings may later ignore or disable them. In both domains, the feedback can improve measurement and action, but it can also create selection effects, label leakage, missing-not-at-random data, or a misleading appearance of model improvement.

Engineering control of this loop requires more than periodic model retraining. A deployment record should distinguish acquisition drift, model drift, user-induced change, and outcome-mediated change; log sensor placement, interface version, threshold changes, missingness, and relevant user actions; monitor calibration and error under the resulting strata; and define when to recalibrate, retrain, roll back, defer, or require human review. Retraining should be evaluated against a stable reference set and consequential human outcomes so that adaptation to behavior is not mistaken for improved sensing. These provisions turn behavioral feedback from a general observation into a monitorable AI-sensor requirement.

### 3.6. Three Sub-Domains of the Perception–Interaction Layer

Within the proposed organizing framework, the perception–interaction layer is divided into three sub-domains according to signal origin and the type of human perceptual transformation involved. The first is *exteroceptive cyber-sensing*, which concerns the cyber-mediated extension or reconstruction of external senses such as sight, hearing, touch, smell, and taste. The second is *interoceptive cyber-sensing*, which concerns the sensing, interpretation, and feedback of internal bodily states. The third is *machine-native cyber-sensing*, which concerns modalities that humans do not naturally possess, such as radar, LiDAR, infrared, ultrasound, magnetic sensing, hyperspectral imaging, or distributed environmental sensing.

The terms *exteroceptive* and *interoceptive* are borrowed from physiology and perception research, where they distinguish information about the external environment from information about the body’s physiological condition [36,37]. Cyberism adapts them to classify technologically mediated perceptual relations rather than neural pathways. Earlier Cyberism descriptions used the label “extrasensory” for the third domain; this review uses *machine-native* because it states the engineering distinction directly and avoids parapsychological connotations.

These three sub-domains differ in their design questions. Exteroceptive systems ask how a human sense can be enhanced, substituted, or reconstructed. Interoceptive systems ask how internal bodily states can be externalized without replacing bodily self-awareness. Machine-native systems ask how nonhuman signals can be translated into forms that humans can understand, trust, and govern.

Table 2 gives a more operational distinction. It is intended to reduce conceptual ambiguity between the three sub-domains. For example, a tactile interface and a radar interface may both involve external objects, but they belong to different sub-domains because the former reconstructs a human sense while the latter translates a nonhuman sensing modality.

The distinction is analytic rather than rigid. Many practical systems are hybrid, and their Cyberism classification can change as the interface changes. An augmented-reality headset that overlays object labels on a camera view is primarily exteroceptive when it supports visual recognition, but it becomes machine-native when it overlays thermal, radio-frequency, or structural information that humans cannot directly perceive. A rehabilitation robot with tactile actuators and physiological monitoring combines exteroceptive and interoceptive mediation. A smart vehicle that displays a camera image, radar confidence, and predicted pedestrian intention combines all three.

#### Decision Workflow for Hybrid Systems

For a hybrid system, the classification can be made with five practical steps: (1) identify the intended human recipient and the perceptual object offered to that recipient; (2) list each signal path and its AI transformation; (3) assign a dominant sub-domain and record secondary sub-domains rather than forcing a one-label description; (4) select the primary construct and the secondary failure checks from the case matrix; and (5) record the degradation, equity, contestability, and deferral provisions that follow from the selected relation. If no human recipient or human-affected decision can be identified, the system should be reported as outside the human-centered scope or as a machine-to-machine boundary case.

In a brain–computer interface, the physiological origin of the neural signal does not by itself determine the dominant sub-domain. Classification follows the perceptual object and intended human action. A decoded control signal that is translated into cursor movement may create a machine-native-to-exteroceptive hybrid relation, whereas feedback that represents fatigue, arousal, or another bodily state raises an interoceptive relation.

An industrial inspection system becomes machine-native when thermal, acoustic, or hyperspectral signals are translated into an operator’s actionable defect or safety judgment. The relevant unit is the human-facing transformation, not the sensor label.

The taxonomy is tested below with three hardware-constant contrasts. The first uses one augmented-reality headset equipped with a visible-light camera and a thermal imager. Registered labels on ordinary visual objects support exteroceptive extension, whereas a thermal risk overlay supports machine-native translation. The second uses one smart-ring inertial measurement unit (IMU). Gesture control with visual or haptic confirmation is an exteroceptive interaction or, if no perceptual output returns to the user, a boundary case outside the taxonomy; sleep or rest-state interpretation is interoceptive. The third uses one in-cabin RGB or near-infrared camera. Gaze-based pointing extends visual interaction, whereas fatigue or attention-state feedback externalizes a body-related state. These contrasts do not claim that hardware alone establishes a category. They test whether changing the perceptual object changes the design record and the human outcome measure. These comparisons and their resulting records are summarized in Table 3.

Hybrid cases are not a weakness of the framework. They are precisely where the framework becomes useful. Engineering taxonomies often separate sensors by modality, while users experience the system as one perceptual situation. The Cyberism question is therefore: which perceptual relation should guide the design priority? If the main risk is confusing a tactile cue with natural touch, perceptual coherence is usually primary. If the main risk is making users anxious about internal signals, interoceptive agency is usually primary. If the main risk is asking humans to rely on a machine-native inference, perceptual explainability is usually primary. The qualifiers matter because secondary constructs remain relevant, as the matrix below makes explicit.

The three sub-domains also suggest different failure modes. Exteroceptive systems can fail through perceptual mismatch, such as latency, unnatural mapping, or feedback that conflicts with motor intention [6]. Interoceptive systems can fail through measurement error or over-interpretation, such as presenting noisy physiological estimates as decisive health meaning [7,38]. Machine-native systems can fail through sensor faults, adversarial interference, or opacity that limits human oversight [39,40]. These failure modes are not captured fully by accuracy, precision, recall, or root-mean-square error. They require evaluation at the level of human interpretation and action.

### 3.7. A Three-Level Methodological Agenda

Cyberism does not ask sensor researchers to abandon engineering rigor. It asks that engineering rigor be connected to human-level and philosophical-level questions. We propose a three-level methodological agenda.

The first level is engineering and technology. It includes transducer design, signal conditioning, embedded inference, sensor fusion, uncertainty modeling, energy-aware edge AI, and system integration [1,2]. This level answers whether the sensing system works. It is the foundation of any AI sensor.

The second level is empirical human study. It includes psychophysics, human factors, user experience research, clinical validation, field studies, trust calibration, and behavioral evaluation [26,27]. This level answers how humans perceive, interpret, and act on the system. It is especially important when AI sensors provide feedback to users rather than only data to machines.

The third level is philosophical and normative reflection. It includes questions of autonomy, responsibility, meaning, fairness, privacy, dignity, and human flourishing, all of which recur across AI ethics frameworks [28]. This level answers what kind of human–cyberspace relationship the system makes possible or difficult. It is not a decorative afterthought. It can shape engineering requirements, such as the need for explainability in autonomous perception or the need for controllable feedback in health wearables.

In practical research design, the three levels can be used as a staged checklist. At the engineering level, researchers specify the signal chain, model assumptions, sensor placement, calibration procedure, uncertainty source, and edge deployment constraints. At the empirical level, they specify who will use the perceptual output, what action the output supports, how trust or confusion will be measured, and whether performance changes over repeated use. At the philosophical and normative level, they specify whether the system shifts responsibility, creates dependence, excludes certain users, or changes what counts as reliable perception. The checklist does not require every paper to become a social-science study. It asks authors to make explicit which parts of the human–cyberspace relation are in scope and which remain open.

This staged method also helps connect laboratory validation with field deployment. In the laboratory, sensor systems are often tested under controlled noise, known ground truth, and short interaction windows. In the field, the same systems encounter ambiguous contexts, motion artifacts, device-specific error, sensor drift, changing user goals, and degraded environmental conditions [38,39]. A Cyberism-informed evaluation should therefore report not only model performance but also the stability of the perceptual relation over time. For a haptic system, this may include adaptation curves, perceived ownership of feedback, or conflict between artificial cues and natural proprioception [6]. For a wearable system, it may include alert fatigue, self-efficacy, reflective use, or anxiety; evidence from patients with atrial fibrillation indicates that wearable use can be associated with increased symptom monitoring, health-care use, and anxiety in response to rhythm notifications [29]. For an autonomous perception system, evaluation may include explanation timing, recoverability after perception failure, and the ability of human supervisors to contest or override machine perception.

The three levels are mutually constraining. A normative requirement such as autonomy preservation can imply an engineering requirement for adjustable feedback intensity, local data processing, or transparent uncertainty bands. An empirical finding such as user over-reliance can imply a redesign of model confidence communication [26,27]. A technical limitation such as sensor drift can imply a need for interface-level humility, where the system explicitly indicates when perception is degraded [38,39].

In practice, the agenda is evaluated in short, separable records. The engineering record states the signal chain, operating limits, and calibration. The human-study record states the recipient, task, outcomes, and repeated-use window. The normative record states who can contest the output, who bears error, and which uses require deferral. This separation makes the proposed method easier to audit and prevents philosophical claims from being mistaken for experimental findings. Figure 3 represents this iterative relationship.

### 3.8. Illustrative Case Mapping

The following cases apply the mapping protocol by identifying the dominant signal source, the human perceptual channel affected, the AI function, and the resulting human–cyberspace relation. The analysis is intentionally compact so that the cases illustrate the method without turning the paper into a domain survey.

#### 3.8.1. Haptic Feedback and Exteroceptive Cyber-Sensing

*Engineering anchor and perceptual transformation.* Haptic systems demonstrate how AI-enabled sensing can reconstruct a classical external sense. Touch is not a single signal channel. It includes pressure, vibration, shear, temperature, texture, proprioception, and affective contact. Tactile perception depends on mechanoreceptors, skin mechanics, motor action, and expectations [6,13,41]. Electronic skin and wearable haptics detect or render tactile information through compliant materials, distributed sensors, actuators, and feedback algorithms [42,43,44]. Recent work in neuromorphic tactile perception further shows that tactile sensing can be encoded through event-like or spike-like representations inspired by biological touch [3].

*Primary failure mode.* From the Cyberism perspective, haptics is not simply sensory substitution. It is sensory reconfiguration. A technically faithful force or vibration can still conflict with motor intention, arrive too late, or feel spatially displaced. The design target is therefore perceptual coherence for a specific body, task, and context. Reviews of artificial touch and wearable haptics likewise emphasize that effectiveness depends on modality, timing, placement, perception, and task demands rather than a single fidelity measure [6,44].

*Evaluation implication.* Multimodal haptic systems should be evaluated not only by signal-level alignment, such as force reconstruction error or temporal synchronization, but also by perceived coherence, measured through task error, response time, subjective integration, and adaptation over repeated use. The corresponding design priority is the human-perceived relation between action and feedback, not only the technical similarity between physical and rendered stimuli.

#### 3.8.2. Wearable Health Sensors and Interoceptive Cyber-Sensing

*Engineering anchor and perceptual transformation.* Wearable health sensors externalize body-coupled signals as graphs, alerts, scores, and recommendations [7,45]. A wrist photoplethysmography (PPG) system illustrates the chain: optical measurements affected by contact and motion are processed into heart-rate estimates; an algorithm compares the estimate with a threshold or personal baseline; and the interface presents a trend or alert that may prompt rest, remeasurement, or help-seeking. AI can add anomaly detection, prediction, personalization, and decision support. Reviews of wearable and implantable biosensors describe movement toward continuous, multimodal, AI-integrated monitoring while identifying motion artifacts, device-dependent accuracy, data quality, privacy, interpretability, and clinical adoption as continuing challenges [38,46,47].

*Primary failure mode.* The central issue is not only whether the wearable measures correctly, but how measurement changes bodily self-awareness, understood against interoception as the sensing and integration of signals originating within the body [45]. External indicators can support self-management by redistributing information-processing demands, as described in cognitive-offloading research [16]. They can also encourage over-reliance when a noisy alert is treated as decisive or when a user discounts felt symptoms because a dashboard appears normal. Evidence from wearable cardiac monitoring further shows that notifications can be associated with symptom monitoring, health-care use, and anxiety, making the interpretation of alerts part of the design problem rather than an incidental response [29].

*Evaluation implication.* Health sensor interfaces should reinforce, rather than replace, interoceptive agency. A wearable should help users ask, “What does this signal mean in relation to how I feel, what I did, and what I should do next?” Evaluation should combine engineering measures, including signal quality, activity-stratified error, and false-alert burden, with human measures, including felt-state–feedback calibration, appropriate remeasurement or help-seeking, self-efficacy, anxiety, alert fatigue, and longitudinal behavior. A testable question follows: do interfaces that combine physiological data with reflective prompts and uncertainty ranges improve self-regulation and appropriate response compared with interfaces that present only scores and alerts?

For PPG, acquisition validity should be reported before interpretation of a component-level indicator or observed user outcome. The acquisition audit should prespecify potential factors including skin-pigmentation-related optical variation, peripheral perfusion, contact pressure, temperature, device fit, activity, signal quality, calibration, and subgroup-specific missingness, together with the conditions for remeasurement or deferral; it should not treat every listed factor as directly established by a single study [38].

Component-level outcomes should be interpreted as evidence about interoceptive agency only after acquisition validity, model calibration, and interface interpretability have been established. When acquisition quality is unequal across bodies or conditions, adverse outcomes should first be treated as potential system-level sensing-validity problems rather than as evidence of reduced user agency. The construct is therefore not redefined by PPG bias; in this framework, any inference about interoceptive agency is conditioned on demonstrated acquisition validity, model calibration, and interface interpretability. When these conditions are not met, the observation is attributed first to the sensing pipeline rather than to the user’s agency.

#### 3.8.3. Autonomous Driving Perception and Machine-Native Cyber-Sensing

*Engineering anchor and perceptual transformation.* Autonomous driving systems integrate cameras, LiDAR, radar, ultrasonic sensors, inertial measurement units, maps, and vehicle-to-everything communication. Public datasets and benchmarks have accelerated progress in object detection, tracking, prediction, and scene understanding [48,49]. Recent peer-reviewed surveys identify camera–LiDAR, camera–radar, bird’s-eye-view (BEV)-centric fusion, cross-modal attention, and multimodal learning as major approaches to robust autonomous perception [8].

*Primary failure mode.* Radar and LiDAR are not improved human vision; they are machine-native channels translated by AI into object lists, occupancy grids, trajectories, risk estimates, and driving actions. A technically accurate detection may still be difficult for an operator, passenger, regulator, or pedestrian to interpret. The problem becomes acute when sensing is degraded by interference, miscalibration, adverse environmental conditions, component faults, or physical adversarial manipulation [39,40].

*Evaluation implication.* Autonomous perception should be evaluated not only by whether the system detected an object, but also by whether a relevant human can understand the basis, uncertainty, and action implications of the detection. Experiments on semi-autonomous driving explanations and vehicle-to-pedestrian intent communication provide empirical anchors: interface choices can affect understanding, trust, performance, and perceived safety, with effects depending on audience and context [30,31]. Perceptual explainability does not require exposing every model parameter. It requires translating machine-native perception into reasons that support the recipient’s task.

A testable research question follows: does presenting perception uncertainty as spatially organized evidence, such as risk heatmaps or sensor-contribution overlays, improve human supervisors’ ability to detect perception failures compared with a single confidence score? Candidate outcomes include failure detection accuracy, intervention reaction time, appropriate override behavior, and calibrated trust after degraded sensing events. Table 4 summarizes the many-to-many relation between the three sensing sub-domains and the evaluation constructs.

The matrix prevents the cases from being treated as a bijection. A wearable alert, for example, may require perceptual explainability as well as interoceptive agency, and a radar-derived haptic warning may require perceptual coherence as well as explanation. The dominant construct sets the first evaluation burden; secondary cells identify foreseeable failure pathways that should be included when the interface or use context activates them. The engineering translation of the three illustrative AI-sensor chains is compared in Table 5.

#### 3.8.4. Worked Crosswalk: A Human-Supervised Multimodal Sensing Scenario

To make the operational claim concrete, consider one analytical scenario: a human-supervised mobility and safety system that presents a visible-light camera cue, a wearable PPG estimate of the human supervisor’s physiological state, and a radar or thermal risk inference during the same decision context. The three paths need not share one physical enclosure; they share a human recipient who may continue, slow, or intervene. This is a constructed crosswalk from the documented signal chains above, not a report of a deployed system or a new experiment [8,38,39].

A modality- or task-only description would label the components as camera, PPG, and radar (or as perception and monitoring) and could apply one common accuracy or confidence reporting logic. That description records the hardware but leaves the human-facing perceptual object and the corresponding failure and oversight burden unspecified. Applying the present protocol yields three linked but non-interchangeable records:

1. The visual path is exteroceptive when a camera cue extends visual recognition. Its primary evaluation burden is perceptual coherence: spatial registration, timing, and compatibility with the supervisor’s action, rather than detection accuracy alone [48,49].
2. The PPG path is interoceptive when it externalizes a bodily state as a trend, score, or alert. Its primary burden is interoceptive agency: acquisition validity, model calibration, uncertainty, and the option to remeasure or defer; a poor signal must not be read as reduced user agency [29,38].
3. The radar or thermal path is machine-native when it translates a modality without an ordinary human equivalent into a risk or occupancy representation. Its primary burden is perceptual explainability: sensor contribution, degradation, evidence meaning, and authorized override, rather than a final confidence score alone [8,31,39].

To make the contrast explicit, suppose the baseline applies one common confidence or alert rule to all three outputs. It could route a low PPG estimate as evidence that the supervisor is less able to intervene and treat a high radar or thermal score as sufficient without a channel-specific degradation or override record. For this constructed scenario, both decisions are wrong at the level of failure attribution: the first confounds acquisition validity with agency, and the second hides the conditions under which machine-native evidence should be trusted or deferred. The crosswalk rejects those defaults and therefore changes the design and oversight record [31,38,39].

The crosswalk forces a concrete design choice: the system should not collapse the three outputs into one undifferentiated confidence or alert channel. Visual feedback should be checked for spatial and temporal coherence; PPG feedback should expose acquisition quality, uncertainty, and remeasurement or deferral; radar or thermal feedback should expose channel degradation and an authorized intervention path. A modality/task baseline can name the components, but it does not by itself determine these distinct provisions. This is the sense in which the crosswalk can change a design and oversight record; it is not a claim that every conventional taxonomy empirically produces the wrong decision.

The worked case also specifies a falsification test. If a prespecified comparison of the modality/task baseline with the crosswalk produces the same failure analysis, measurement bundle, and oversight provisions, the framework’s incremental value is not established. Such a comparison would require an empirical study with a defined task, recipient, degradation conditions, and decision outcomes; the present review supplies no participant data or performance estimate.

### 3.9. Cross-Case Synthesis

The cases share a signal-to-inference-to-representation architecture but diverge in the human-facing transformation. Haptic systems must coordinate artificial feedback with ongoing action; wearables must relate computed indicators to felt state and self-management; autonomous perception must translate evidence that an unaided person cannot directly inspect. Accuracy, latency, robustness, and calibration remain necessary, but a single usability score would conceal these distinct failure pathways.

Time and audience also differ. Haptic incorporation can require repeated exposure because mapping and adaptation affect whether a cue becomes part of motor control [6,43,44]. Wearable effects can accumulate through alerts, checking, reassurance, avoidance, or altered device use, so short-term comprehensibility must be separated from longer-term self-efficacy, anxiety, adherence, and help-seeking [7,29,38]. In autonomous systems, drivers, passengers, remote supervisors, pedestrians, investigators, and regulators require different evidence at different times [30,31]. Perceptual explainability is therefore recipient- and task-specific.

Uncertainty can originate in transduction, preprocessing, models, interfaces, or human interpretation. The intervention must match the source: placement or shielding cannot repair model shift, retraining cannot repair an unintelligible display, and a confidence score cannot repair a failed sensor. A minimum evidence bundle should therefore report signal and model validity under relevant conditions, recipient comprehension and action, foreseeable degradation, repeated-use effects, and differential performance across relevant users and settings [25,50].

The comparison also creates a falsification route. If the three constructs reduce to one undifferentiated usability factor, or if the taxonomy does not improve prediction of failure and intervention needs beyond established sensor or task categories, it should be revised. Evidence that different transformations require distinguishable measure bundles would support discriminant value without validating every broader claim of Cyberism.

## 4. Discussion

### 4.1. From Accuracy Optimization to Perceptual Experience Design

The first implication of the Cyberism perception–interaction layer is a shift from accuracy optimization to perceptual experience design. Accuracy remains essential, but it is not sufficient. Accuracy measures the gap between a system output and a physical or labeled reference. Perceptual experience design asks a different question: whether the user can integrate the sensed and inferred information into action, trust, and self-understanding. Automation research shows that technically capable systems can still produce misuse, disuse, or inappropriate reliance when human understanding and trust are poorly calibrated [26,27].

This distinction has practical consequences. A wearable electrocardiogram sensor with strong arrhythmia detection performance can still fail at the level of use if notifications increase anxiety or encourage dependence on alerts rather than bodily awareness [29]. A high-fidelity haptic system can still feel unnatural if its feedback timing, modality, or placement conflicts with action and perception [6,43,44]. An autonomous vehicle with strong object detection performance can remain socially opaque if surrounding humans cannot understand its perception, uncertainty, or intention, especially under degraded or adversarial sensing conditions [31,39,40]. In all three cases, the sensor may measure correctly while the human–cyberspace relation fails.

Perceptual experience design therefore addresses the full loop. It considers the signal, the model, the interface, the user’s interpretation, and the behavioral response that changes future sensing. For exteroceptive systems, designers should ask whether artificial feedback can be incorporated into bodily action without excessive cognitive translation. For interoceptive systems, they should ask whether the interface strengthens self-regulation rather than replacing felt experience with scores. For machine-native systems, they should ask whether nonhuman channels become understandable enough for oversight, trust calibration, and contestability [27,30,31].

Perceptual experience design therefore requires new evaluation criteria. For exteroceptive systems, perceptual coherence should be measured alongside resolution and latency. For interoceptive systems, interoceptive agency, understood here as a domain-specific form of somatic agency, should be measured alongside prediction accuracy. For machine-native systems, perceptual explainability should be measured alongside detection and tracking metrics. These criteria are not soft substitutes for engineering metrics. They are system-level outcomes that determine whether engineering performance becomes meaningful in human use.

### 4.2. Equity in Cyber-Mediated Sensing

The second implication concerns differential performance and access in cyber-mediated sensing. The evidence cited here plays bounded roles. Gender Shades documents intersectional performance disparities in commercial gender classification, not a complete human-facing sensing loop [51]. Bent et al. report device- and activity-dependent optical heart-rate error under one study protocol, not a universal skin-tone effect [38]. Pulse-oximetry studies compare sensor readings with arterial measurements and document clinically consequential racial and ethnic discrepancies, but the devices studied are not themselves evidence that an AI feedback loop produces the same disparity [52,53]. Together, these studies justify a reporting requirement; they do not validate a new equity construct or one mechanism shared by all AI sensors.

Reporting should separate four layers. At acquisition, authors should state relevant transduction mechanisms, placement, missingness, operating conditions, and subgroup coverage. At the model layer, they should report stratified calibration and error when scientifically justified by mechanism and use context. At the interface layer, they should test whether intended recipients can perceive uncertainty, understand recommended action, and contest an inference. At the downstream layer, they should document access to devices, connectivity, follow-up, and the consequences of false reassurance, unnecessary alarm, or deferral. Edge processing can change latency, privacy, and connectivity demands, but its benefits still depend on device capability and deployment resources [1,2].

The proposed constructs organize rather than prove this reporting. Perceptual coherence can reveal whether a feedback mapping or device placement works differently across bodies and contexts; interoceptive agency asks whether external indicators support or displace judgment for different users; and perceptual explainability asks whether recipients can recognize degraded sensing and obtain an actionable account. Equity is therefore a cross-cutting requirement applied to each construct and system layer, with hypotheses and subgroup variables prespecified from the sensing mechanism rather than inferred after observing a difference [28].

The same logic applies to institutions. AI risk-governance guidance treats roles, accountability, documentation, human review, incentives, and lifecycle monitoring as conditions of responsible deployment, while health-AI guidance links governance to public benefit, equity, and protection from harm [54,55]. In a sensor deployment, procurement rules, reimbursement or insurance arrangements, vendor incentives, maintenance budgets, workplace authority, liability allocation, and access to follow-up care can change who receives a device, which errors are tolerated, how alerts are escalated, and whether a person can contest an output. These institutional pathways are part of the closed loop because they shape deployment, feedback, and the consequences of error. Fairness analysis should therefore record not only subgroup performance but also the pathway through which a signal becomes actionable or ignored.

### 4.3. AI-Mediated Perception and Human Oversight

The third implication is that interpretation increasingly begins inside the sensing loop. The model filters, labels, ranks, predicts, and sometimes acts before a human sees the signal, consistent with established accounts of automation mediating human information acquisition and action [25,26]. AI may therefore be described metaphorically as a co-perceiver, but the framework does not attribute consciousness or human perception to a model. The precise claim is that AI inference structures the information available for human perception and action.

This mediated role changes design responsibilities. Model uncertainty becomes a perceptual feature, not only a statistical parameter. Data provenance becomes part of warranted reliance, not only part of system documentation. Explanations become part of the perceptual interface, not only post-hoc compliance artifacts. Human-centered AI and visual-analytics research emphasizes communicating system capabilities, uncertainty, and changes over time, and relating uncertainty awareness to calibrated trust [25,27,50]; Cyberism locates such communication within the broader human–cyberspace relation and asks where meaningful human oversight remains possible.

Oversight must also be located in time. Before deployment, oversight includes selecting sensors, defining operating limits, evaluating subgroup performance, and deciding which outputs require human confirmation. During use, it includes recognizing degraded sensing, understanding the action supported by an inference, and having enough time and authority to intervene. After an event, it includes reconstructing which signals, transformations, and interface states shaped the decision. A system can offer extensive documentation after the fact while providing little actionable oversight at the moment of risk. Conversely, a real-time warning can support intervention but remain insufficient for accountability if its evidence cannot later be reconstructed.

The degree of automation does not by itself determine whether oversight is meaningful. A manual interface can overwhelm a user with poorly organized sensor data, while a highly automated system can preserve oversight through clear operating boundaries, calibrated alerts, and recoverable control. The relevant question is whether responsibility, information, and practical authority remain aligned. Human-automation research shows that asking a person to supervise a capable automated system can produce misuse or disuse when capabilities and limits are poorly understood [26,27]. The Cyberism lens extends this concern to the perceptual evidence on which supervision depends.

### 4.4. Construct Boundaries and Validation Logic

The three core constructs are intended to organize evaluation, not to function as interchangeable labels for good user experience. *Perceptual coherence* concerns integration between artificial feedback, bodily expectation, and action. *Interoceptive agency* concerns the capacity to use externalized bodily information while retaining self-regulation and judgment. *Perceptual explainability* concerns task-relevant understanding of machine-native sensing, uncertainty, and likely action. All three can influence trust or workload, but neither trust nor workload defines any one of them.

Discriminant cases clarify these boundaries. A haptic cue may be easy to explain verbally yet remain perceptually incoherent because it arrives late or conflicts with movement. A wearable display may be visually clear and technically accurate yet weaken interoceptive agency if users treat its score as overriding symptoms or context. An autonomous interface may feel smooth and natural yet offer poor perceptual explainability if users cannot recognize which sensor has degraded or predict the next system action. These cases show why a general usability measure cannot stand in for the proposed constructs.

The constructs also differ from neighboring established measures. Garfinkel et al. distinguish interoceptive accuracy, measured by objective behavioral performance; interoceptive sensibility, measured through subjective report; and interoceptive awareness, defined as metacognitive correspondence between confidence and accuracy [56]. MAIA and broader interoception research assess additional dimensions of noticing, interpreting, regulating, and trusting bodily sensations [45,57]. Interoceptive agency does not replace these constructs. It adds a sensor-mediated conflict and action condition: whether a person can integrate, question, and appropriately act on an external representation of bodily state while retaining judgment.

Perceptual explainability is also narrower than XAI and distinct from situation awareness. XAI taxonomies classify model transparency and post-hoc explanation techniques and ask how explanations should be evaluated for a purpose and audience [32,33]. Situation awareness models address perception, comprehension, and projection in dynamic environments [34]. Perceptual explainability focuses on the link between sensed evidence and human oversight: can a specified recipient detect degradation, understand relevant uncertainty, anticipate likely system action, and contest or override it when authorized? Trust in automation and NASA-TLX remain secondary measures because reliance and workload can change without demonstrating this capability [27,58,59].

Validation should therefore proceed from content and task definition to convergence and discrimination. Content validity requires domain experts and intended users to agree that the proposed tasks cover the relevant perceptual relation. Behavioral indicators should then be examined alongside self-report rather than replaced by it. Convergent evidence would show expected associations with related constructs, such as interoceptive awareness or calibrated trust. Discriminant evidence would show that the new construct predicts relevant outcomes after accounting for generic usability, workload, or confidence. Known-groups and experimental-degradation comparisons can test sensitivity, while repeated measurements can test adaptation and stability.

Failure criteria must be specified as clearly as success criteria. Perceptual coherence is not supported when subjective naturalness rises but task conflict or error worsens. Interoceptive agency is not supported when engagement increases through compulsive checking, anxiety, or indiscriminate deference. Perceptual explainability is not supported when an interface raises confidence without improving prediction, failure detection, or appropriate intervention. These asymmetric criteria are important because a persuasive display can improve subjective ratings while degrading oversight.

Construct validation is also domain-bound. A haptic coherence task validated for teleoperation cannot be assumed valid for prosthetic touch, rehabilitation, or virtual reality. An interoceptive-agency task developed for cardiac alerts may not transfer unchanged to glucose, sleep, stress, or fertility monitoring. An explanation that supports a trained remote operator may be inappropriate for a passenger or pedestrian. Claims should therefore name the population, task, sensor, degradation conditions, and time horizon to which the evidence applies.

### 4.5. Design Principles and Testable Hypotheses

The proposed Cyberism framework can be translated into concrete design actions. First, designers should specify the intended perceptual relation before selecting a sensor architecture or model family: extension of an existing sense, externalization of bodily state, or translation of a machine-native modality. This statement can prevent a mismatch in which the technical pipeline is optimized without a clear account of the human perceptual role it will serve.

Second, interfaces should communicate uncertainty in a task-relevant form. In AI sensing, uncertainty is usually represented internally through probabilities, confidence scores, calibration curves, or Bayesian estimates. For human users, however, uncertainty is encountered through doubt, reassurance, alarm, and decisions about reliance. Research on uncertainty awareness and trust supports treating uncertainty communication as part of the user-facing analytic process [27,50]. A wearable may need to distinguish trend, anomaly, and measurement noise. A driving system may need to distinguish confirmed objects from weakly inferred risks. A haptic interface may need to signal delayed or degraded feedback. The design question is not only whether uncertainty is computed but whether it supports the user’s decision.

Third, feedback should preserve adjustment, contestability, and human judgment when people remain responsible for action. This is especially important in health, mobility, work safety, and assistive technologies. Preserving agency does not mean minimizing automation. It means enabling users to understand, contest, adjust, or learn from it, consistent with research on appropriate reliance and recurring AI ethics principles of autonomy, transparency, and accountability [26,27,28]. Concrete provisions include adjustable thresholds, layered explanations, confidence-aware alerts, manual override, and reflective summaries that connect sensor output to context.

These principles generate hypotheses suitable for empirical study. For exteroceptive cyber-sensing, one hypothesis is that, at matched physical fidelity, a coherent feedback mapping will reduce task error under feedback conflict relative to an incoherent mapping. For interoceptive cyber-sensing, a second hypothesis is that interfaces presenting a physiological signal with uncertainty and contextual reflection will improve appropriate remeasurement or help-seeking relative to score-only feedback. Self-regulation and alert fatigue will be evaluated as secondary outcomes, while anxiety, indiscriminate deference, and compulsive checking will be prespecified as safety or behavioral guardrails. For machine-native cyber-sensing, a third hypothesis is that sensor-contribution uncertainty displays will improve failure-detection sensitivity relative to final-output confidence scores. Behavior prediction, intervention time, appropriate override, and trust calibration will be secondary outcomes; false alarms on nominal trials and trust increases without better intervention will be prespecified as oversight guardrails.

The hypotheses also suggest measurement strategies. Perceptual coherence can be assessed through task performance, adaptation speed, perceived naturalness, and conflict between artificial feedback and bodily expectation. No validated single instrument currently measures *interoceptive agency* as defined here. It should therefore be evaluated as a multi-method construct using calibration tasks that compare felt state with sensor feedback, behavior under deliberately conflicting feedback, domain-specific self-efficacy and regulation behavior, and longitudinal anxiety, alert fatigue, adherence, or help-seeking. The Multidimensional Assessment of Interoceptive Awareness (MAIA) provides relevant self-report subscales, including self-regulation, body listening, and trusting, but can support only convergent assessment rather than directly validate the proposed construct [57]. Perceptual explainability can be assessed through user prediction of system behavior, recovery after error, failure detection, and appropriate override or reliance. Established trust-in-automation scales can provide a secondary measure of trust calibration, while the NASA Task Load Index (NASA-TLX) can measure workload; neither instrument alone measures perceptual explainability [58,59]. These measures should complement, not replace, conventional AI sensor metrics. Table 6 summarizes the operational definitions, measurement bundles, and current measurement maturity of the three constructs.

A non-significant result alone will not be treated as falsification. Interpretation will distinguish evidence of a practically meaningful benefit, insufficient precision, evidence that the upper confidence limit for benefit lies below the prespecified minimum, and violation of a safety or behavioral guardrail. The relevant effect thresholds and decision rules must be specified before data collection [60].

Table 7 makes the evidentiary status explicit. Each hypothesis names a manipulation, a primary outcome, and an outcome pattern that would count against the proposed construct. No hypothesis licenses a numerical result in the present conceptual review.

The practical value of this translation is that Cyberism becomes more than a descriptive vocabulary. It becomes a way to design experiments, compare interface alternatives, and report human-centered implications in AI sensor papers. A manuscript can state its sub-domain, identify the perceptual relation under study, report technical metrics, and then add one or two human–cyberspace measures aligned with that relation. Over time, this reporting habit could help the intelligent-sensing community build cumulative knowledge about how intelligent sensing systems reshape perception.

For interoceptive agency in particular, instrument development should precede claims of construct validation. A staged program would first establish content validity with users, clinicians, and sensor researchers; then test factor structure, internal consistency, and test–retest reliability; and finally assess convergent and discriminant validity against interoceptive awareness, health anxiety, self-efficacy, and objective calibration tasks. Measurement invariance should be examined across relevant device types, age groups, health contexts, and skin-tone groups before group comparisons are interpreted. Until such evidence exists, studies should report the component measures separately rather than combine them into an unvalidated total score.

### 4.6. Partial Instruments and New Experimental Paradigms

The three constructs differ in measurement maturity. Perceptual coherence has partial task-level instruments: timing, task error, adaptation, psychophysical discrimination, and perceived naturalness can be measured now, but their bundle remains domain-specific. Interoceptive agency has partial components: MAIA, objective interoceptive accuracy, confidence calibration, self-efficacy, anxiety, and alert-fatigue measures can be combined as convergent evidence, but no validated instrument directly measures the proposed sensor-mediated construct. Perceptual explainability also has partial components: comprehension, failure detection, behavior prediction, trust calibration, override, and workload can be measured, but no generic XAI score can substitute for a task-specific human-oversight paradigm.

New paradigms are required when the question concerns conflict between sensed and felt state, recognition of source degradation, or action under uncertainty. These paradigms should manipulate the relevant signal or interface condition, define ground truth or reference standards, and report the component outcomes separately before any latent construct is proposed. This distinction prevents the construct table from being read as evidence that a complete validated battery already exists.

### 4.7. Proposed Empirical Validation Protocol

The framework can be tested without treating this conceptual review as completed empirical research. The following protocol is a preregistration-ready proposal, not a report of collected data. It targets the machine-native sub-domain because the worked thermal and autonomous-driving examples make the competing interface choices explicit.

In a supervised driving or hazard-inspection simulation, participants would encounter nominal sensing and controlled degradation, such as occlusion, calibration drift, or loss of one sensor channel. The primary contrast would be a sensor-contribution uncertainty display versus a single final-output confidence score; a spatial risk map with uncertainty would be an exploratory secondary condition. The physical sensor inputs, model, event set, and decision thresholds would remain constant across conditions. Condition order would be counterbalanced, and manipulation checks would verify that participants noticed the display and degradation cue.

For each prespecified degraded or failed sensing event, the trial-level outcome would be a binary hit/miss indicator: a hit occurs when the participant correctly flags the event within the prespecified decision window. Failure-detection sensitivity is the detection probability across event-present trials, not a statistic defined by a single trial. The primary estimand would be the between-condition difference in standardized marginal detection probability on the absolute-risk-difference scale. Secondary endpoints would include correct prediction of the system’s next action, intervention time, appropriate override, trust calibration across accurate and degraded trials, decision confidence, and workload measured with NASA-TLX [59]. If a self-report trust scale is used, the selected instrument and its role as a secondary measure should be prespecified [58]. Nominal trials would be used to estimate the false-positive rate and specificity, with an acceptable absolute limit or between-condition comparison rule specified before data collection. Additional exploratory comparisons would be labeled as such; any claim based on multiple confirmatory contrasts would use a prespecified multiplicity-control procedure.

A logistic mixed-effects model would be fitted to the trial-level hit/miss outcome. The specification would account for participant-level repeated observations and scenario-level clustering; random intercepts for participant and scenario would be the minimum structure, with a prespecified random slope for condition when supported by the design. The analysis plan would prespecify the target covariate and scenario distribution and the treatment of random effects used to obtain standardized marginal detection probabilities. The absolute risk difference and its 95% confidence interval would be computed using a prespecified model-based standardization or participant-level bootstrap procedure. Power and precision simulations would target this same estimand and estimation procedure [61]. If a continuous replacement endpoint is used, a linear mixed-effects model with corresponding participant and scenario structure would be specified as a separate analysis rather than substituted after data collection.

Sensitivity and specificity would be reported as paired summaries across event-present and nominal trials. Signal-detection summaries such as d-prime or criterion may be added when the design prespecifies signal and noise events and false alarms are part of the estimand; these summaries should not replace the primary model or alter the endpoint after data collection [62].

For planning, investigators should preregister one primary contrast, a two-sided alpha of 0.05, and a target power of at least 0.80 (0.90 when missed failures have safety-critical consequences). For the proposed binary endpoint, the default precision target is a 95% confidence interval with maximum half-width 0.10 on the absolute-risk-difference scale; for a continuous replacement endpoint, the maximum acceptable half-width of the 95% confidence interval should be 0.20 standard deviations. Domain-specific thresholds may replace these defaults only with a written rationale before data collection.

The estimand must be stated on the outcome scale. For the binary primary endpoint, the model-standardized marginal absolute risk difference would be the primary effect and the odds ratio a secondary model-scale effect; both would be reported with 95% confidence intervals. The prespecified planning effect for the binary endpoint is an absolute difference in failure-detection sensitivity. In the absence of a documented domain-specific or operational threshold, investigators may use an absolute increase of 0.10 as a provisional planning anchor; this value is not an empirical result or a universal clinical threshold and must be replaced or justified before data collection when a domain-specific threshold is available. This effect-size anchor is separate from the precision target above. For a continuous replacement endpoint, the mean difference and standardized mean difference would be reported with 95% confidence intervals. A standardized mean difference of 0.40 may be used only as a sensitivity-analysis anchor for a continuous replacement endpoint when no domain-specific threshold or pilot estimate exists.

*No-fixed-N simulation template: define N as the number of participants recruited; choose candidate recruitment sizes; simulate attrition, participant and scenario effects, repeated trials, event rates or outcome variance, and the prespecified subgroup composition within each candidate N; fit the prespecified mixed-effects model and apply the prespecified marginal standardization procedure; record power, bias, and confidence-interval half-width over B replicates; and select the smallest N meeting both the power and precision targets. Do not apply an additional inflation for attrition, repeated measures, clustering, or subgroup composition when those factors are already represented in the simulation.*

The study report should state the assumed effect scale, event rate or variance, correlation structure, number of repeated trials, attrition mechanism, clustering, subgroup plan, number of simulation replicates, random seed, convergence handling, confidence-interval-width decision rule, and exact simulation or power-analysis code. These details turn the protocol into a reproducible planning rule while preserving the fact that the present paper contains no participant data.

Evidence for perceptual explainability would require more than higher self-reported trust. The strongest pattern would combine better failure detection and behavior prediction with appropriate rather than indiscriminate intervention, without an unacceptable workload increase. A format that merely raises trust during degraded sensing would count against the construct. Replication across the exteroceptive and interoceptive domains would then test whether the three-part taxonomy predicts genuinely different outcome bundles rather than relabeling one general usability effect.

### 4.8. A Cyberism-Driven Research Agenda for AI-Enabled Sensing

Figure 4 summarizes a research agenda for AI-enabled sensing. Three directions correspond to the core evaluation constructs. The first is perceptual coherence: aligning sensor fusion and feedback with human perceptual integration. The second is interoceptive agency: designing wearables that strengthen self-understanding instead of replacing it. The third is perceptual explainability: translating machine-native perception into humanly meaningful reasons. A cross-cutting fourth direction is equity in cyber-mediated sensing: evaluating whether access, performance, and oversight differ across users and settings.

This agenda also suggests reporting practices. Authors of AI sensor papers can add a short human–cyberspace interpretation paragraph: what perceptual channel is transformed, what AI function mediates it, what human agency issue may arise, and what evaluation beyond accuracy is appropriate. Such a paragraph would not overburden technical papers, but it would make the human implications of sensing more explicit.

#### Engineering Reporting Examples

A conventional wearable-sensing paper could add one paragraph stating that its PPG estimate is an interoceptive externalization, naming activity-stratified acquisition limits, the uncertainty shown to users, the recommended remeasurement action, and the risk of false reassurance or alert fatigue. It would then report the technical error metrics together with the relevant human outcome and subgroup checks.

A conventional industrial-vision paper could add one paragraph stating that a thermal or hyperspectral defect score is machine-native translation for an operator. It would identify which evidence the operator can inspect, how calibration drift or channel loss is signaled, what intervention is authorized, and how disagreement with the model is recorded. This is a compact addition to an engineering report, not a claim that the paper has conducted a new human study.

For engineering teams, this reporting practice can be implemented as a lightweight design record. At the beginning of a project, the record would specify the intended perceptual relation, the expected user action, the uncertainty that must be communicated, and the conditions under which the sensor should defer, warn, or remain silent. During evaluation, the same record can be revisited to compare technical performance with the actual human response. This makes Cyberism actionable without requiring every AI sensor study to adopt a large philosophical apparatus.

### 4.9. Toward Cumulative Evidence Across AI Sensor Domains

A research agenda becomes useful only when individual studies can be compared. At present, sensor papers often report rich technical metrics while human studies report task-specific experiences using different terminology. The proposed framework offers a minimal common index: sub-domain, signal source, AI transformation, human-facing representation, intended action, degradation condition, technical outcome, human outcome, and agency or equity concern. Reporting these elements would not standardize away domain differences; it would make those differences visible enough for later synthesis.

Evidence can accumulate through staged maturity rather than one universal validation study. An early-stage prototype may establish that a perceptual mapping is detectable and learnable. A controlled experiment can compare mappings, explanation formats, or uncertainty displays under defined degradation. A field study can examine adaptation, fatigue, dependence, and changing goals over time. Clinical or safety-critical deployment requires additional validation against domain ground truth, consequential outcomes, and relevant populations. Normative and governance analysis should accompany these stages by identifying who can contest the system, who bears error, and which uses should remain outside the system’s authority.

Cross-domain comparison should focus on relations rather than raw scores. A one-second response time has different significance in a wearable reflection task, a haptic motor-control loop, and a driving intervention. Likewise, trust is not uniformly beneficial: greater trust is appropriate only when it tracks demonstrated capability and degradation. Meta-analytic comparison would therefore require harmonized task definitions and outcome directions before pooling. Until such harmonization exists, structured qualitative comparison and replication of specific interface contrasts are more defensible than a single composite Cyberism score.

Equity should be embedded in this cumulative program rather than added after technical validation. Relevant subgroup variables depend on the sensing mechanism and use context; they may include skin tone for optical sensing, body morphology and sensor placement, disability, age, language, expertise, device quality, connectivity, or access to follow-up care. The existence of one documented disparity does not justify assuming all others, but it does justify prespecifying plausible sources of differential performance and reporting when the sample cannot test them [38,51,52,53]. Where a difference is observed, studies should separate signal acquisition, model behavior, interface interpretation, and downstream access so that remedies target the responsible layer.

Finally, cumulative evidence requires explicit correction and stopping rules. A construct should be narrowed when it cannot be measured reliably, a sub-domain boundary should be revised when hybrid systems are classified inconsistently, and a design recommendation should be withdrawn when it improves subjective appeal without improving appropriate action. These rules keep the agenda empirical even though the present paper is conceptual. They also prevent the framework from becoming self-confirming: future evidence must be able to show when Cyberism terminology adds no predictive or practical value.

### 4.10. Limitations

This paper has limitations. First, it proposes a conceptual framework and a validation protocol but reports no empirical validation. The categories of exteroceptive, interoceptive, and machine-native cyber-sensing should be tested across more domains, including environmental sensing, industrial inspection, smart homes, affective computing, and assistive technologies. Second, the literature synthesis is purposive rather than systematic, and the case studies are illustrative rather than exhaustive; neither supports prevalence estimates or claims of comprehensive coverage. Third, the framework is intentionally human-centered, which means it may require extension for ecosystems in which nonhuman agents, robots, or distributed infrastructures are the primary users of sensing outputs. Fourth, Cyberism is itself an emerging doctrine; its terminology, construct measures, and boundaries will need refinement through use by sensor engineers, HCI researchers, clinicians, ethicists, and social scientists. The integrated crosswalk demonstrates possible analytical decision divergence, but it does not establish incremental validity against a conventional sensor or task taxonomy. A preregistered comparative study remains necessary to determine whether the proposed crosswalk improves failure analysis, measurement selection, or oversight in practice.

## 5. Conclusions

AI-enabled sensing is becoming a primary interface between humans and cyberspace. This article introduced the perception–interaction layer of Cyberism as a framework for understanding and designing that interface. The central argument is simple: intelligent sensors should be evaluated not only by what they measure, but also by what perceptual relation they create.

The paper identified three sub-domains. Exteroceptive cyber-sensing extends and reconstructs external senses; interoceptive cyber-sensing externalizes and interprets internal bodily states; and machine-native cyber-sensing translates nonhuman modalities into humanly actionable perception. These sub-domains clarify why haptic feedback, wearable health sensing, and autonomous driving perception raise different human–cyberspace questions even when all are technically AI sensor systems.

The proposed three-level methodological agenda links engineering design, empirical human study, and philosophical reflection. It supports a practical shift from accuracy alone to the three core evaluation constructs of perceptual coherence, interoceptive agency, and perceptual explainability, while treating equity in cyber-mediated sensing as a cross-cutting requirement. The integrated worked crosswalk and proposed validation protocol show how the framework can change interface choices and generate falsifiable comparisons without claiming uncollected evidence.

Cyberism should therefore be understood not as a replacement for sensor engineering but as an emerging analytical and normative lens on the human meaning of sensor engineering. In the present context, its value lies in asking a question that technical metrics alone cannot answer: when AI sensors mediate perception, what kind of human–cyberspace relation are they building? This narrower claim remains open to refinement through comparative scholarship and empirical validation.

## Figures and Tables

**Figure 1:**
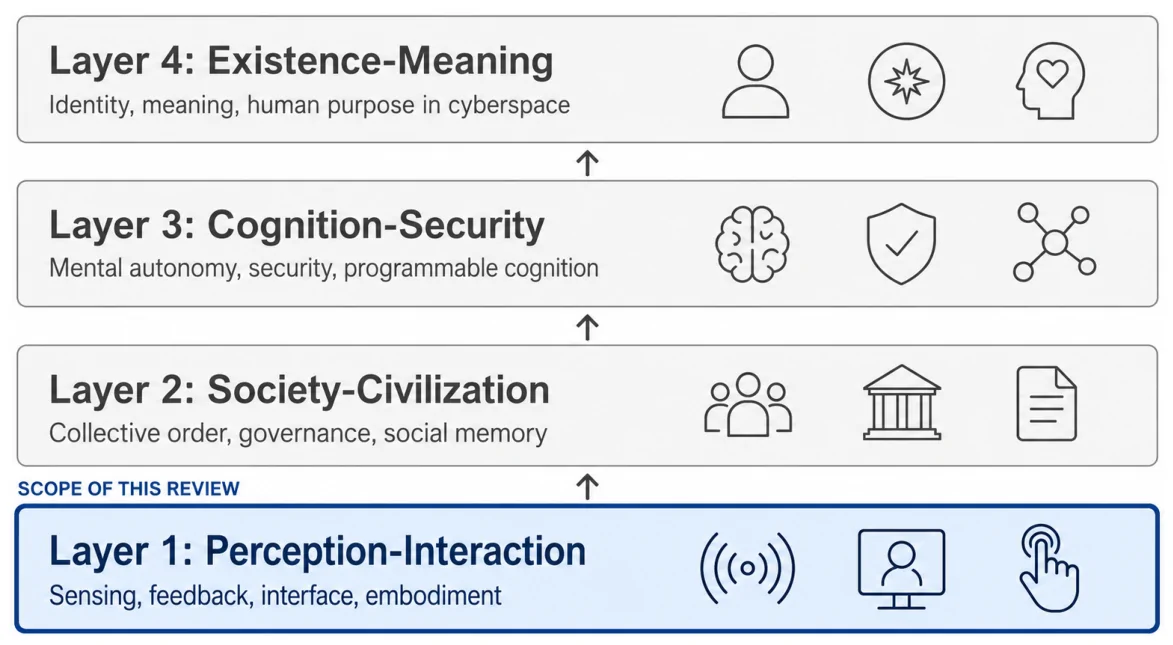
Cyberism as a four-layer framework for human–cyberspace relationships. This review analyzes only the foundational perception–interaction layer, where AI-enabled sensing becomes the interface of embodied cyber experience; the upper three layers are shown solely to locate that layer within the broader framework.

**Figure 2:**
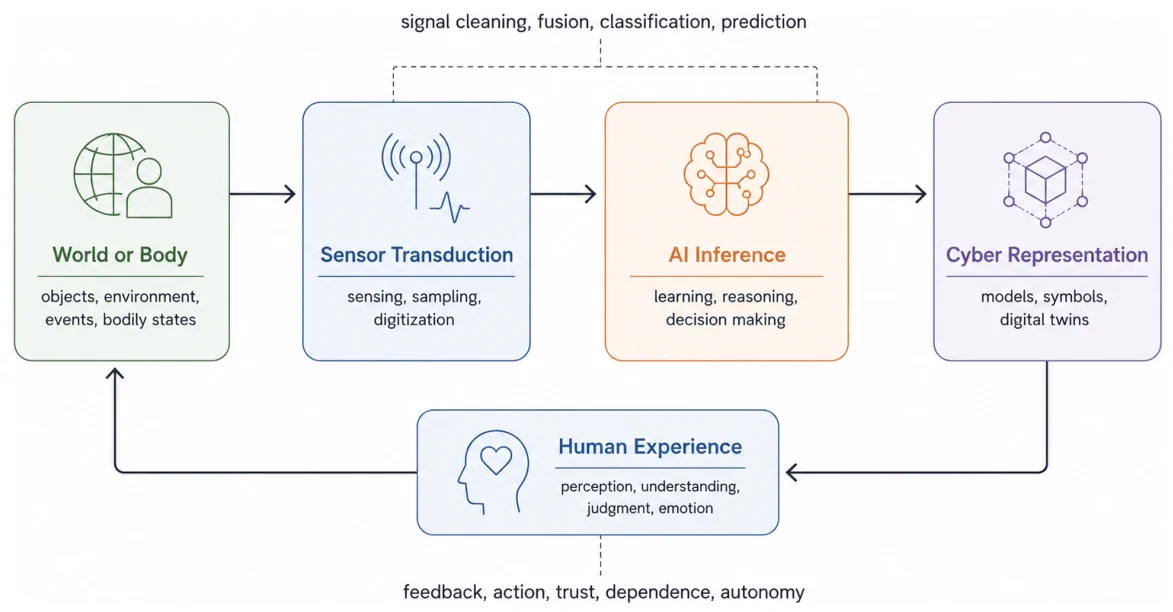
AI-enabled sensing as a human–cyberspace loop. Cyberism highlights the full loop from signal capture to AI inference, cyber representation, human experience, and behavioral feedback.

**Figure 3:**
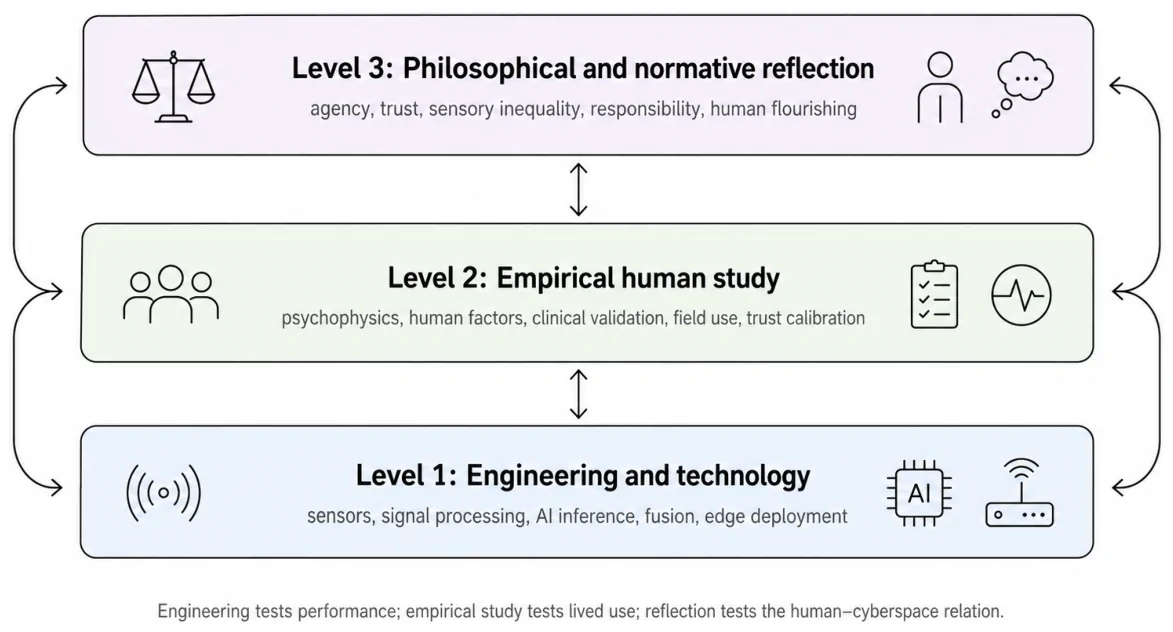
Three-level methodological agenda. Cyberism links technical design, empirical human evaluation, and normative reflection into one iterative process.

**Figure 4:**
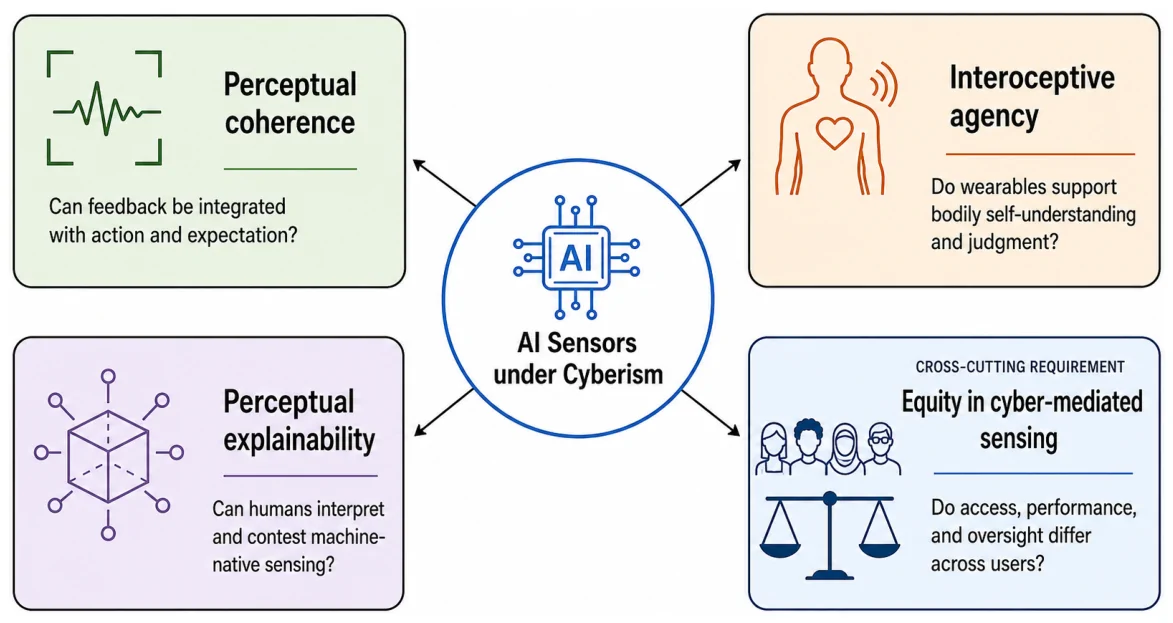
Cyberism-driven research agenda for AI-enabled sensing. The first three panels represent the core evaluation constructs; equity in cyber-mediated sensing is shown as a cross-cutting requirement rather than a fourth core construct.

**Table 1:** Positioning of the Cyberism perception–interaction layer against adjacent frameworks and fields.

Framework or Field	Primary Analytic Axis	Typical Question	Cyberism Complement
Sensor engineering	Signals, devices, models, and edge systems	How accurately and efficiently can the system measure and infer?	Specifies the perceptual relation created by measurement and inference
Postphenomenology	Embodiment, hermeneutic, alterity, and background relations	How does technology mediate human experience of the world?	Classifies the signal origin and perceptual transformation within AI sensing
Sensory substitution and augmentation	Cross-modal remapping and extension of capability	Which sensory capacity is substituted, restored, or augmented?	Distinguishes familiar-sense extension from machine-native translation
Soma design and embodied HCI	First-person bodily experience and design practice	How can interactive systems be designed through and for felt experience?	Links embodied experience to AI functions, uncertainty, and evaluation criteria
AI ethics and governance	Fairness, privacy, accountability, and safety	What norms should guide intelligent systems?	Identifies equity risks, agency shifts, and perceptual oversight

**Table 2:** Operational distinction among three sub-domains of the perception–interaction layer.

Dimension	Exteroceptive Cyber-Sensing	Interoceptive Cyber-Sensing	Machine-Native Cyber-Sensing
Signal source	External world	Internal bodily state	External world or system state captured through machine-native channels
Human perceptual type	Existing external senses	Bodily self-awareness and physiological feeling	Perception humans do not naturally possess
Core AI task	Enhancement, substitution, reconstruction, compression	Filtering, prediction, personalization, interpretation	Fusion, translation, uncertainty estimation, explanation
Design challenge	Perceptual coherence between natural and artificial feedback	Avoiding over-reliance on external indicators and anxiety amplification	Making nonhuman sensing understandable and governable
Representative sensors	Vision sensors, microphones, e-skin, tactile arrays	ECG, EEG, PPG, IMU, glucose, sweat and skin conductance sensors	LiDAR, radar, thermal, ultrasound, hyperspectral and distributed sensor networks

**Table 3:** Hardware-constant classification tests and filled design records. Each row holds the sensing hardware constant while changing the human-facing perceptual role.

Hardware and Paired Use	Dominant Relation	Design Record that Changes	Human Outcome and Failure Test
Visible-light and thermal AR headset: registered visual labels versus thermal risk overlay	Exteroceptive extension versus machine-native translation	Registration, visual-search integration, and action–feedback latency versus temperature scale, calibration state, threshold, uncertainty, and defer/warn rule	Visual-search time and registration error versus hazard detection, recognition of degraded sensing, and appropriate inspection action
Smart-ring IMU: gesture control with command confirmation versus sleep or rest-state interpretation	Exteroceptive interaction or boundary case versus interoceptive externalization	Gesture vocabulary, command confirmation, and interaction latency versus wear-state quality, temporal aggregation, uncertainty, and remeasurement guidance	Selection error and confirmation time versus reference agreement, false-alert burden, checking behavior, and appropriate response
In-cabin RGB/NIR camera: gaze-based pointing versus fatigue or attention-state feedback	Exteroceptive interaction versus interoceptive externalization	Gaze calibration, target mapping, and response latency versus operating limits, confidence communication, nuisance-alert control, and override	Target-selection error and time versus state-detection validity, warning comprehension, appropriate response, and alert avoidance

**Table 4:** Many-to-many relation between sensing sub-domains and evaluation constructs. “Primary” marks the usual leading burden, not an exclusive correspondence.

Sub-Domain	Perceptual Coherence	Interoceptive Agency	Perceptual Explainability
Exteroceptive	**Primary**: timing, spatial mapping, modality fit, and integration with action	**Context-dependent**: relevant when feedback changes bodily confidence, effort, or self-regulation	**Secondary**: needed when inferred or fused cues cannot be checked directly through the familiar sense
Interoceptive	**Secondary**: agreement or conflict between displayed feedback, felt state, and action	**Primary**: capacity to integrate, question, and act on externalized bodily information	**Secondary**: understanding inference, uncertainty, thresholds, and appropriate response
Machine-native	**Secondary**: learnability and stability of the human-facing encoding or feedback	**Context-dependent**: relevant when machine-native evidence changes bodily or behavioral self-regulation	**Primary**: task-relevant understanding of evidence, degradation, uncertainty, and likely action

**Table 5:** Engineering translation of the three illustrative AI-sensor chains. Quantitative values are reported only where a cited study supplies a commensurable device, condition, and reference standard.

Case	Transduction and AI Operation	Artifact or Operating Limit	Human Representation and Failure	Evidence and Monitoring Requirement
Haptic feedback	Force, pressure, vibration, or skin deformation; detection, estimation, mapping, or control	Sensor/actuator dynamics, placement, latency, saturation, and body/task dependence	Vibrotactile, force, or skin-stretch cue; conflict with movement or spatial expectation	Reviews identify timing, placement, modality, and task dependence but no universal error range [6,44]; report task-specific timing, amplitude, detection, conflict, and adaptation
Wearable PPG	Optical blood-volume change; filtering, heart-rate estimation, anomaly detection, or personalization	Prespecified potential acquisition factors include skin-pigmentation-related optical variation, peripheral perfusion, contact pressure, temperature, motion artifact, activity, device fit, device algorithm, interpolation, and missingness; not all factors are established by the cited study	Trend, score, or alert; false reassurance, nuisance alert, compulsive checking, or displaced bodily judgment	Bent et al. report an ECG-referenced study of 53 participants in which MAE ± SD was 7.2 ± 5.4 bpm at rest and 10.2 ± 7.5 bpm during activity for consumer devices, and 13.9 ± 7.8 and 15.9 ± 8.1 bpm for research-grade devices; use this as direct evidence for the reported comparison, while the other listed factors remain prespecified audit factors requiring direct evidence in future studies [38]. Interpret interoceptive-agency outcomes conditionally on acquisition validity and model calibration.
Autonomous perception	Camera, LiDAR, radar, ultrasound, or IMU; calibration, detection, fusion, tracking, and prediction	Weather, occlusion, interference, miscalibration, channel loss, dataset shift, and adversarial perturbation	Object, occupancy, trajectory, risk, or action; hidden degradation or inappropriate reliance	Fusion and failure surveys document architecture- and scenario-dependent limits but no universal cross-modality error range [8,39,40]; report per-modality validity, calibration, missing channels, scenario strata, and recovery

**Table 6:** Operational definitions, measurement bundles, and current measurement maturity for the three core evaluation constructs.

Construct	Operational Definition	Primary Indicators	Measurement Bundle	Current Measurement Maturity
Perceptual coherence	Degree to which artificial feedback can be integrated with bodily action and expectation without persistent conflict	Task error, response time, adaptation rate, perceived naturalness	Task-specific performance and ratings, adaptation curves, and sensor–action timing	Partial and domain-specific; no validated universal measure
Interoceptive agency	Capacity to integrate sensor feedback with felt bodily states while retaining self-regulation and judgment	Felt-state calibration, self-efficacy, anxiety, appropriate response, behavior change	Calibration and conflict tasks plus self-efficacy and longitudinal outcomes; MAIA supports convergent assessment, not direct validation	Partial components only; no validated direct instrument for the sensor-mediated construct
Perceptual explainability	Capacity to predict, interpret, and appropriately contest machine-native sensing outputs	Failure detection, behavior prediction, reaction time, appropriate override or reliance	Comprehension and failure-detection tasks; trust scale and NASA-TLX are secondary measures, not direct validation	Partial components only; task-specific validation is required

**Table 7:** Core testable hypotheses and proposed preregistration-ready decision and guardrail criteria. These are proposals for future empirical work, not results of the present review.

ID	Sub-Domain	Planned Contrast	Primary Outcome	Decision and Guardrail Criterion
H1	Exteroceptive	Matched physical fidelity: coherent versus incoherent feedback mapping	Task error under feedback conflict	Unfavorable if the upper 95% confidence limit for error reduction is below the prespecified minimum, or if error or adaptation time worsens
H2	Interoceptive	Signal plus uncertainty and reflection versus score-only feedback	Appropriate remeasurement or help-seeking response	Unfavorable if the upper 95% confidence limit for the appropriate-response benefit is below the prespecified minimum, or if deference or compulsive checking exceeds a guardrail
H3	Machine-native	Sensor-contribution uncertainty display versus final-output confidence score	Failure-detection sensitivity	Unfavorable if the upper 95% confidence limit for the sensitivity benefit is below the prespecified minimum, or if either false alarms or trust without better intervention exceeds a guardrail

## Data Availability

No new data were created or analyzed in this study. Data sharing is not applicable to this article.

## Abbreviations

The following abbreviations are used in this manuscript:

AI	Artificial intelligence
AIoT	Artificial intelligence of things
BEV	Bird’s-eye view
CPST	Cyber-physical-social-thinking
ECG	Electrocardiography
EEG	Electroencephalography
HCI	Human–computer interaction
IMU	Inertial measurement unit
IoT	Internet of things
LiDAR	Light detection and ranging
PPG	Photoplethysmography

## Appendix A. Premise-to-Evidence Audit

**Table A1:** Premise-to-evidence audit of the three central premises. The audit records what remains of each premise when Cyberism terminology is removed.

Premise	Non-Cyberism Evidence	Alternative Classification	Negative or Boundary Case	Design or Measurement Consequence	Decision/Status
P1: human-facing perceptual object	Haptics, wearable sensing, and explainable driving studies describe how signals become feedback, alerts, or action-relevant representations [6,30,38]	A conventional sensor-type or task taxonomy can classify the component without specifying what a person is meant to perceive	Machine-to-machine output with no identifiable human recipient; or a camera stream reduced to a machine-only latent representation	Record the intended recipient, perceptual object, and human action; classify only when the human-facing relation changes a design or measurement decision	Retained; narrowed to human-facing systems
P2: distinct human failure modes	Haptic timing conflict, optical-sensor error and automation misuse show that different signal-to-feedback transformations can create different failure pathways [6,26,38]	Generic usability or technical-error categories would treat these as one undifferentiated performance problem	The same hardware supports different mappings; high technical accuracy is accompanied by inappropriate reliance or poor action	Prespecify the dominant failure mode, secondary checks, and the human outcome that would count against the mapping	Retained as a falsifiable analytic premise; not an empirical universal
P3: evaluation beyond device/model performance	Human–automation, uncertainty-communication, and AI-risk-governance work links technical performance to reliance, oversight, documentation, and accountability [26,50,54]	An accuracy-only benchmark or sensor taxonomy would omit recipient understanding, intervention, and institutional consequences	A high-confidence output improves a technical metric but leaves failure detection, contestability, or appropriate intervention unchanged	Report technical metrics with task-relevant human outcomes, degradation checks, uncertainty communication, and governance records	Retained as a normative and measurement requirement; not a validated outcome
